# Thin filament dysfunctions caused by mutations in tropomyosin Tpm3.12 and Tpm1.1

**DOI:** 10.1007/s10974-019-09532-y

**Published:** 2019-07-03

**Authors:** Joanna Moraczewska

**Affiliations:** Department of Biochemistry and Cell Biology, Faculty of Natural Sciences, Kazimierz Wielki University in Bydgoszcz, Bydgoszcz, Poland

**Keywords:** Thin filament, Tropomyosin, Cardiomyopathy, Congenital myopathy

## Abstract

Tropomyosin is the major regulator of the thin filament. In striated muscle its function is to bind troponin complex and control the access of myosin heads to actin in a Ca^2+^-dependent manner. It also participates in the maintenance of thin filament length by regulation of tropomodulin and leiomodin, the pointed end-binding proteins. Because the size of the overlap between actin and myosin filaments affects the number of myosin heads which interact with actin, the filament length is one of the determinants of force development. Numerous point mutations in genes encoding tropomyosin lead to single amino acid substitutions along the entire length of the coiled coil that are associated with various types of cardiomyopathy and skeletal muscle disease. Specific regions of tropomyosin interact with different binding partners; therefore, the mutations affect diverse tropomyosin functions. In this review, results of studies on mutations in the genes *TPM1* and *TPM3*, encoding Tpm1.1 and Tpm3.12, are described. The paper is particularly focused on mutation-dependent alterations in the mechanisms of actin-myosin interactions and dynamics of the thin filament at the pointed end.

## Introduction

The thin filaments of striated muscle contain actin polymers coated with tropomyosin (Tpm) and regularly spaced troponin (Tn). In the sarcomere, the thin filaments are oriented with one end (fast polymerizing or barbed end) anchored in the Z-disc and the other end (slowly polymerizing or pointed end) directed towards the M-line, which is located in the center of the sarcomere. Interactions of the oppositely oriented thin filaments with bipolar myosin thick filaments generate contraction. The crucial factor determining contractile force in striated muscle myofibrils is the number of myosin heads forming active cross-bridges between actin and myosin filaments. This depends on the access of myosin heads to myosin-binding sites on actin, which is tightly controlled by the tropomyosin and troponin complex in response to fluctuations of Ca^2+^ concentration in the sarcoplasm due to muscle cell stimulation. This is a classic function of tropomyosin-troponin that is described by the well-studied steric-blocking and cooperative mechanisms (Gordon et al. 2000; Lehrer and Geeves 1998).

Force production also depends on the degree to which thin and thick filaments overlap. In different types of muscle fibers, thin filaments have been shown to possess various lengths, which correlate with muscle type (Gokhin et al. 2012; Granzier et al. 1991). Thin filament length is an important element of the contractile characteristics of different types of muscle. Good candidates for length regulators are the giant protein molecule nebulin, which is anchored in the Z-disc and extends along thin filaments, and the homologous pointed end capping proteins—tropomodulin (Tmod) and leiomodin (Lmod) (Fowler and Dominguez 2017; Gregorio et al. 1995). High resolution fluorescence microscopy revealed that nebulin stabilizes much of actin filament length, but does not reach the pointed end (Gokhin et al. 2012). In contrast, tropomyosin extends along the entire length of the thin filament where it interacts with Tmod or Lmod and is involved in the maintenance of the correct length of the thin filament (Gregorio et al. 1995; Tsukada et al. 2010). The mechanism by which tropomyosin executes this function is still not well understood.

**Fig. 1:**
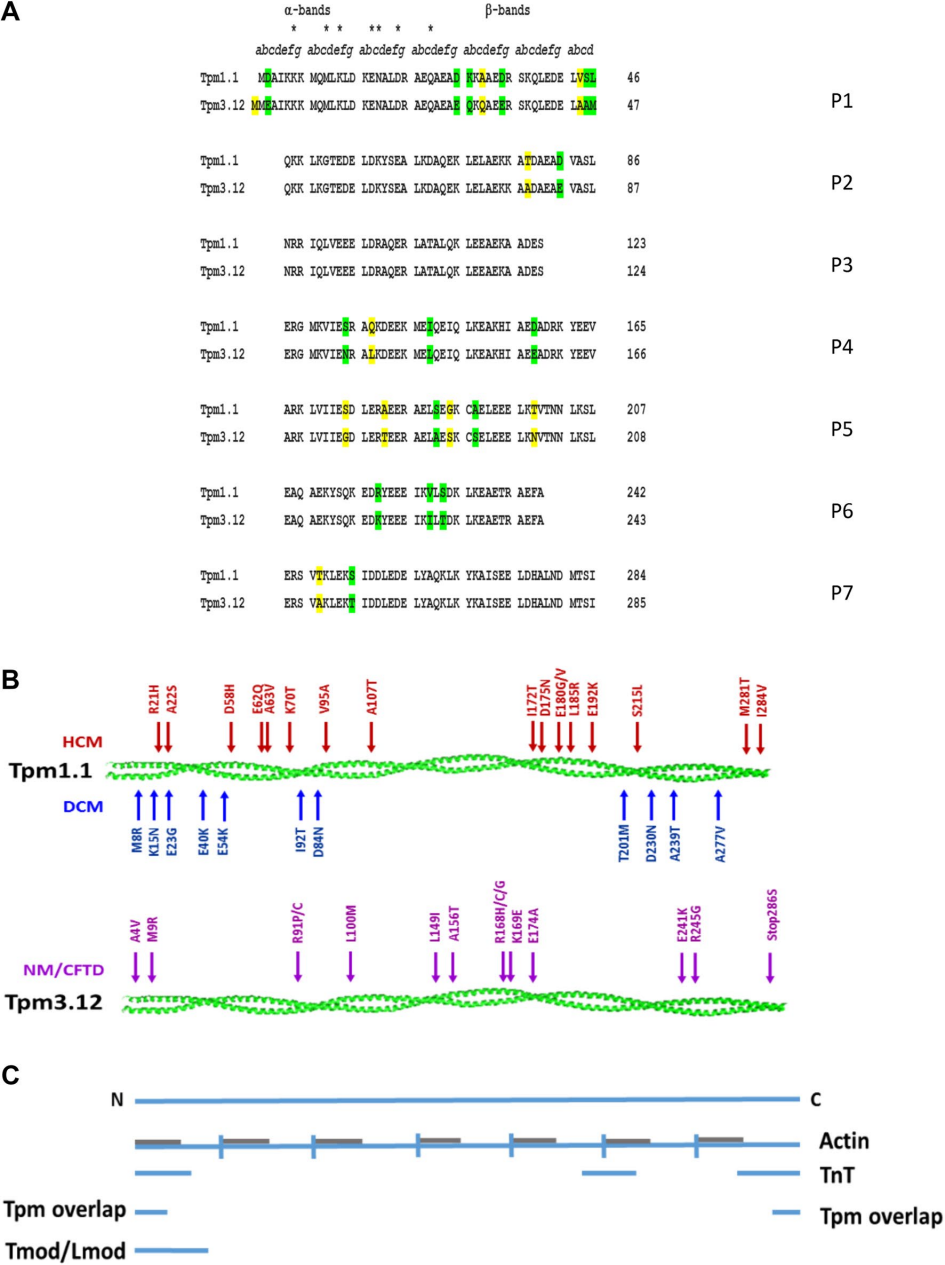
Distribution of differences in amino acid sequences of Tpm1.1 and Tpm3.12 (**a**). Substitutions in Tpm1.1 and Tpm3.12 linked to cardiomyopathy and myopathy-causing mutations in *TPM1* and *TPM3* (**b**). Regions of tropomyosin interactions with proteins of the thin filament (**c**). The alignment of Tpm1.1 and Tpm3.12 sequences with respect to seven actin-binding periods (P1–P7) was adapted from Barua et al. (2011). Conservative and non-conservative substitutions, which distinguish Tpm1.1 from Tpm3.12 are marked in green and yellow, respectively. Amino acid residues corresponding to heptapeptide repeat of the coiled coil (*a*–*g*) are shown on top of the sequences; the consensus sites which potentially interact with residues exposed on actin surface are marked with *. HCM: hypertrophic cardiomyopathy; DCM: dilated cardiomyopathy; NM: nemaline myopathy; CFTD: congenital fiber type disproportion. (Color figure online)

In mammalian cells, about forty different tropomyosin isoforms are expressed (Geeves et al. 2015). Three isoforms are specific for striated muscle—isoforms Tpm1.1 and Tpm2.2 are expressed in all types of striated muscle at various levels, and Tpm3.12 is restricted to slow muscle fibers (Pieples and Wieczorek 2000; Corbett et al. 2005). Detailed analyses of tropomyosin expression in single fibers of different bovine muscle types demonstrated that in large animals Tpm1.1 and Tpm3.12 are found exclusively in fast and slow fibers, respectively (Oe et al. 2016). All three isoforms exist either as homo- or heterodimers (Janco et al. 2013; Jin et al. 2016; Peng et al. 2013). The sequence of human Tpm3.12 differs from Tpm1.1 in 25 amino acids, i.e. they are 91% identical. Because many of the corresponding substitutions are conservative (Fig. 1a), the similarity between Tpm1.1 and Tpm3.12 is actually ~ 96%. However, experimental data strongly suggest that the high similarity of the isoforms is not simply a case of evolutionary redundancy, but has functional meaning. For example, overexpression of Tpm3.12 in hearts of transgenic mice decreases systolic and diastolic function and reduces Ca^2+^ sensitivity of heart muscle fibers (Pieples et al. 2002). Therefore, Tpm1.1 and Tpm3.12 may be evolutionary developed to finely tune the functions of different types of muscles.

Structural and functional diversity of tropomyosin isoforms has been recently described in several excellent reviews (Brettle et al. 2016; Gunning et al. 2015; Hitchcock-DeGregori and Barua 2017; Khaitlina 2015; Manstein and Mulvihill 2016). In this paper, I focus on current knowledge of the functional effects of disease-related mutations in two tropomyosin genes—*TPM1* and *TPM3*, with special regard to the effects of mutations on interactions between proteins, which additionally bind at the pointed end of the filament. The available data not only reveal molecular mechanisms underlying certain types of congenital skeletal muscle and cardiac diseases, but also provide insight to the physiological differences between Tpm1.1 and Tpm3.12.

## Cardiac and skeletal muscle pathology associated with mutations in tropomyosin genes

Missense mutations in *TPM1*, the gene encoding Tpm1.1, are linked to hypertrophic (HCM) and to dilated cardiomyopathy (DCM). In humans, about 30 single-residue mutations distributed along the entire length of tropomyosin are known (Redwood and Robinson 2013). The mutations are specific for the development of either HCM or DCM (Fig. 1b).

HCM is characterized by systolic hypercontractility and impaired relaxation due to left ventricular wall and septal hypertrophy. Studies on isolated muscle fibers as well as on reconstituted thin filaments consistently show that the amino acid substitutions in Tpm1.1 increase sensitivity to Ca^2+^ concentrations. Many mutations accelerate the rate of actin-myosin interactions, decrease relaxation and increase force production. The hallmarks of DCM are increased ventricular chamber and systolic hypocontractility. Most DCM-associated mutations in Tpm1.1 lead to reduced tension and decreased rate of actin-myosin interaction. In contrast to HCM, sensitivity to activating Ca^2+^ concentrations is decreased in DCM [reviewed by (Redwood and Robinson 2013; Wieczorek et al. 2008)].

Point mutations in *TPM3* encoding slow skeletal Tpm3.12 (Fig. 1b) are involved in two known skeletal muscle myopathies: nemaline myopathy (NM) and congenital fiber type disproportion (CFTD). The major traits of NM are: disarray of sarcomeres, hypotrophy of type 1 muscle fibers and the presence of nemaline bodies, which are pathological structures consisting of actin, α-actinin and other proteins normally present in Z-discs. Predominance and hypotrophy of slow, type 1 muscle fibers in the absence of other pathological features such as nemaline bodies is dominant in CFTD, though distortion of the Z-disc can occur. Both conditions present hypocontractile phenotype with various levels of muscle weakness, which correlates with reduced acto-myosin ATPase activation and lower force production in isolated muscle fibers (Clarke 2008; Kee and Hardeman 2008). So far, no mutation in *TPM3* linked to a hypercontractile phenotype has been found.

Different phenotypes observed in Tpm1.1-dependent cardiomyopathy or Tpm3.12-dependent skeletal muscle myopathy strongly suggest that the mutations must cause structural distortions in regions of the tropomyosin molecule, which differentially affect the functions of the thin filament. Even small structural changes can result in large functional aberrations. Because tropomyosin self-polymerizes, and binds F-actin, troponin, Tmod and Lmod (Fig. 1c), localization of the mutation-linked substitutions along the molecule potentially can affect any of these different interactions.

## Structural determinants of tropomyosin functions

### Interactions of tropomyosin with actin

**Fig. 2:**
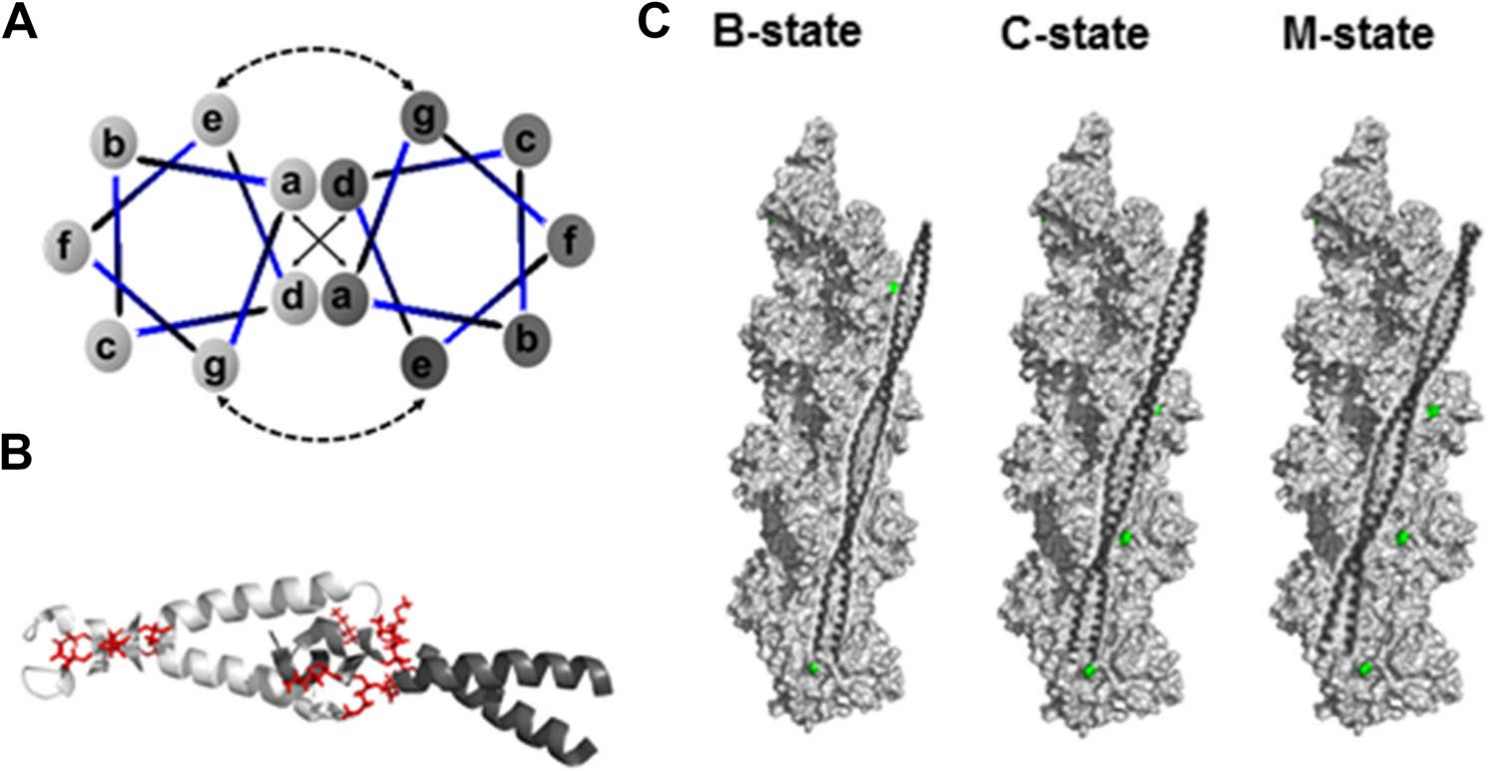
Tropomyosin interactions within the coiled coil (**a**), in the overlap between adjacent tropomyosin molecules (**b**) and with F-actin in the B- C- and M-state (**c**). Coiled-coil stabilization by hydrophobic interactions between *a* and *d* residues at the interface between α-helices and by corresponding electrostatic interactions between *e* and *g* residues within two turns of α-helices (heptapeptide). NMR structure of the C-terminus (light grey) and N-terminus (dark grey) of tropomyosin Tpm1.1 model peptides showing the overlap region of tropomyosin (PDB: 2G9 J); consensus, actin-binding sites (Lys6, Met10, Lys12, Asp254, Asp255, Asp258) are shown in red sticks. For illustration of the azimuthal positions of tropomyosin in the B-, C- and M-state, tropomyosin segment comprising residues 148–284 (PDB: 6bno) was aligned in PyMol on F-actin (PDB: 1c1 g). Pro333 (green spheres) was used as a marker of the boundary between the C- and B-state positions (Kiani et al. 2019). (Color figure online)

Two-chain tropomyosin is stabilized by hydrophobic interactions between residues localized in the core of the coiled coil. The hydrophobic core is formed by a helical fold of a heptapeptide repeat, a sequence of seven amino acids (*a*-*b*-*c*-*d*-*e*-*f*-*g*) with mostly large hydrophobic residues at positions *a* and *d*, which are exposed on one face of each α-helix. When the chains wind around each other, the hydrophobic residues are locked in “knobs into holes” fashion. Electrostatic interactions between residues in positions *e* and *g* provide additional stabilization (Fig. 2a). Although the coiled-coil structure extends along tropomyosin chains, substantial conformational variety is present within the core domain. The canonical pattern of bulky hydrophobic residues within the core is interrupted by small hydrophobic or polar residues, which affect packing and stability along the molecule (Brown et al. 2005; Minakata et al. 2008; Nitanai et al. 2007). Conformational stability has local and long-range effects on tropomyosin bending flexibility, which is required for tropomyosin functions. Refer to the paper by Lehman and colleagues in this special issue (Lehman et al. 2019) for a detailed discussion on the different types of flexibility and their functional consequences. Various aspects of tropomyosin structure/function relationships were also described in several reviews (Hitchcock-DeGregori 2008; Hitchcock-DeGregori and Barua 2017; Hitchcock-DeGregori and Singh 2010; Nevzorov and Levitsky 2011).

Each of seven pseudo-repeating modules in the extended muscle tropomyosin molecule align with one of seven successive actin subunits along thin filaments. This holds for sequences of Tpm1.1 and Tpm3.12 as illustrated in Fig. 1a, were aligned in rows to demonstrate regions comprising seven actin-pseudo-repeating binding periods (P1–P7). Despite being homologous in sequence, the actin-binding periods do not bind to actin with the same affinity (Hitchcock-DeGregori et al. 2002). Each period is divided into N- and C-terminal halves (α- and β-bands) (McLachlan and Stewart 1976). The α-bands harbor charged and hydrophobic amino acids in *b*, *c*, *e* and *f* positions of the coiled coil (marked with asterisks in Fig. 1a). These sites are available for interactions with charged residues exposed on actin (Barua 2013; Hitchcock-DeGregori and Singh 2010; Li et al. 2011). Among amino acid residues which distinguish Tpm1.1 from Tpm3.12, four substitutions are present in consensus sites located in actin-binding period 4 and 6 (Q135 vs. L136, I143 vs. L144, R220 vs. K221 and V227 vs. I228), and several substitutions are in close vicinity of the consensus sites. These differences between Tpm1.1 and Tpm3.12 may be responsible for the differences in actin affinities between Tpm1.1 and Tpm3.12 (Moraczewska, unpublished results). The binding analysis is complex because single tropomyosin molecules bind to isolated sites with low affinity. Binding to contiguous sites, i.e. next to the tropomyosin molecule already bound, is facilitated by end-to-end interactions between neighboring molecules. This promotes cooperative tropomyosin binding and greatly increases overall tropomyosin affinity for actin, which results in formation of continuous tropomyosin cables winding along both sides of actin filament (Wegner 1980). Tropomyosin cables are separated radially from the central axis of actin by 38–40 Å, a distance that is too large for stereospecific contacts between these proteins (Lorenz et al. 1995; Holmes and Lehman 2008; Poole et al. 2006; von der Ecken et al. 2015). Most structural studies show that the main type of interactions that stabilize the contacts between F-actin and tropomyosin are weak electrostatic bonds (Li et al. 2011; Orzechowski et al. 2014; Sousa et al. 2013; von der Ecken et al. 2015).

Structures of the C-terminal model peptides demonstrate that the two α-helical chains splay apart at the C-terminus and the coiled coil of the N-terminal segment inserts between bifurcated C-terminal helices (Fig. 2b). The planes of the neighboring molecules are rotated by 90^o^, which accommodates them in a correct position to form specific contacts between actin-binding periods along subsequent tropomyosins and actin subunits (Greenfield et al. 2006; Minakata et al. 2008). Deleting nine amino acids from either one or both ends drastically reduces actin affinity of tropomyosin isoforms (Moraczewska and Hitchcock-DeGregori 2000; Moraczewska et al. 1999), which supports the view that tropomyosin ends play a crucial role in binding to actin with high affinity and in end-to-end interactions leading to tropomyosin cable formation. It is noteworthy that compared to Tpm1.1, the N-terminus of Tpm3.12 has an additional Met residue and Asp to Glu substitution (Fig. 1a), which may affect the structure of the overlap and position of Tpm3.12 on the filament. High resolution structures of this isoform are not available yet; therefore further studies are needed to verify this hypothesis.

### Binding of troponin to the thin filament

In skeletal and cardiac muscle, each tropomyosin binds Tn complex, a Ca^2+^-binding protein, which allows for regulation of contraction in response to fluctuations in sarcoplasmic Ca^2+^ concentration. TnC, TnI and the C-terminus of TnT form the Ca-sensitive core domain of Tn complex. N-terminal segment of TnT anchors Tn complex to the thin filament through interactions with tropomyosin (Perry 1998). Interactions with TnT increase tropomyosin affinity for actin and are crucial for transmission of the Ca^2+^ signal from the Tn core domain to the thin filament (Jackson et al. 1975; Tobacman et al. 2002). TnT is known to have two binding sites for tropomyosin (Jin and Chong 2010); however, the exact residues of the TnT N-terminus and the interactions with tropomyosin are not known. Several studies suggested that site 1 comprises 39 amino acid residues of the middle TnT region and extends along the head-to-tail junction of tropomyosin (Fig. 1c). Tropomyosin-binding site 2 is probably 25-amino acids long and interacts with the middle region of tropomyosin next to the Tn core domain (Jin and Chong 2010). Few conservative and non-conservative substitutions within this region differentiate Tpm1.1 and Tpm3.12 (Fig. 1a); however, whether these differences affect regulatory properties of Tpm-Tn complex still is not known. The differences might be particularly important in determining specific interactions between tropomyosin and isoforms of troponin present in cardiac, fast and slow skeletal muscles (Sheng and Jin 2014; Sheng and Jin 2016; Wei and Jin 2016).

### The thin filament activation states

Tropomyosin cables fluctuate on the filament to assume different azimuthal positions. When bound to actin alone, the actin-binding periods of skeletal muscle tropomyosin interact with charged residues exposed on actin (Barua et al. 2011; Li et al. 2011). This locates tropomyosin chains on the inner domain of the filament (Lorenz et al. 1995). Tn complex bound in the presence or absence of Ca^2+^ as well as strongly bound myosin heads maintain the filament in equilibrium between three activation states (McKillop and Geeves 1993), which are characterized by different positions of tropomyosin chains (Vibert et al. 1997) (Fig. 2c). Binding of Ca^2+^-free Tn positions tropomyosin on the outer domain of the filament. Binding of Ca^2+^ to TnC shifts the filament into the closed C-state, in which tropomyosin assumes an azimuthal position similar to the position observed in the absence of Tn complex (Lehman et al. 1994; Holmes 1995). Binding of myosin heads allows for the filament transition from the C- to M-state, which is characterized by a shift of tropomyosin cables further towards the inner domain of the filament (Behrmann et al. 2012; Fischer et al. 2016; Holmes 1995; Poole et al. 2006; Risi et al. 2017; Vibert et al. 1997). The M-state is produced when TnC is saturated with Ca^2+^ and myosin binds to actin; however the presence of the fourth activation state (M^−^) is possible (Lehrer 2011). The presence of this active state, which can be induced by strongly bound myosin heads in the absence of Ca^2+^; was proposed based on the observation that in the case of HCM, relaxation is incomplete and residual force is generated even at low Ca^2+^ concentration (Lehrer 2011; Lehrer and Geeves 2014).

Changes in tropomyosin position on the filament in response to binding of Tn (± Ca^2+^) and myosin S1 may be due to a rolling or a shifting mechanism for the tropomyosin transition. As originally proposed by McLachlan and Steward, tropomyosin in the Off state might interact with actin via residues in its so-called α-bands. Upon Ca-activation, tropomyosin might change its rotation by rolling 90^o^ around its coiled-coil axis, which would bring the residues in the β-band into contact with actin (McLachlan and Stewart 1976). Fluorescence quenching experiments performed with thin filaments reconstituted in the presence of tropomyosin tagged with fluorescent 5-hydroxytryptophanes along the sequence showed that the residues located in α-bands were less exposed to solvent in the absence of Ca^2+^ and those in the β-bands were less exposed after Ca^2+^ was bound to TnC. This led the authors to conclude that rolling of tropomyosin cables underlies the mechanism of the Ca^2+^-induced activation of the thin filament (Holthauzen et al. 2004). However, the authors did not consider localized twisting or axial displacement of tropomyosin on actin which might have produced the same effects. In fact, current structural data is consistent with the alternative sliding explanation. In F-actin-tropomyosin models obtained by EM and computational methods, in the absence of either Tn or S1, the tropomyosin cable assumes a position which is biased towards the B-state (Li et al. 2011). Tropomyosin position in the M-state was shown in the pseudo-atomic model of the F-actin-tropomyosin-myosin complex (Behrman et al. 2012). The mechanism of the activating transition from the B- to the M-state was deduced by a comparison of tropomyosin interactions with actin in both states and suggested that tropomyosin may slide obliquely with a concomitant upward shift along the filament, which results in a large azimuthal shift (Behrman et al. 2012).

Differing tropomyosin positions are the basis of the classic steric-blocking mechanism of muscle contraction regulation, in which tropomyosin blocks myosin-binding sites on actin (B-state), partially reveals myosin-binding sites (C-state), or does not interfere with myosin binding (M-state) (Lehman 2016). Because tropomyosin covers the entire thin filament, the positions it assumes might also be important for actin interactions with nebulin or myosin-binding protein C, a possibility that has recently gained experimental support (Lin et al. 2018; Marttila et al. 2014a; Risi et al. 2018).

### Regulation of interactions at the pointed end of the thin filament

Specific interactions within the head-to-tail junction reinforce the polarity of each tropomyosin molecule, with the N- and C-terminus oriented towards the pointed and the barbed end of the filament, respectively (Fig. 2c). Consequently, the N-terminal region of tropomyosin is available for interactions with the pointed end-binding proteins—Tmod and Lmod. The importance of these interactions is exemplified by the fact that the presence of tropomyosin on the filaments increases actin affinity of four Tmod and three Lmod isoforms expressed in vertebrate species (Almenar-Queralt et al. 1999; Boczkowska et al. 2015; Chereau et al. 2008; Kostyukova et al. 2007; Rao et al. 2014; Skwarek-Maruszewska et al. 2010; Tsukada et al. 2010; Weber et al. 1994).

**Fig. 3:**
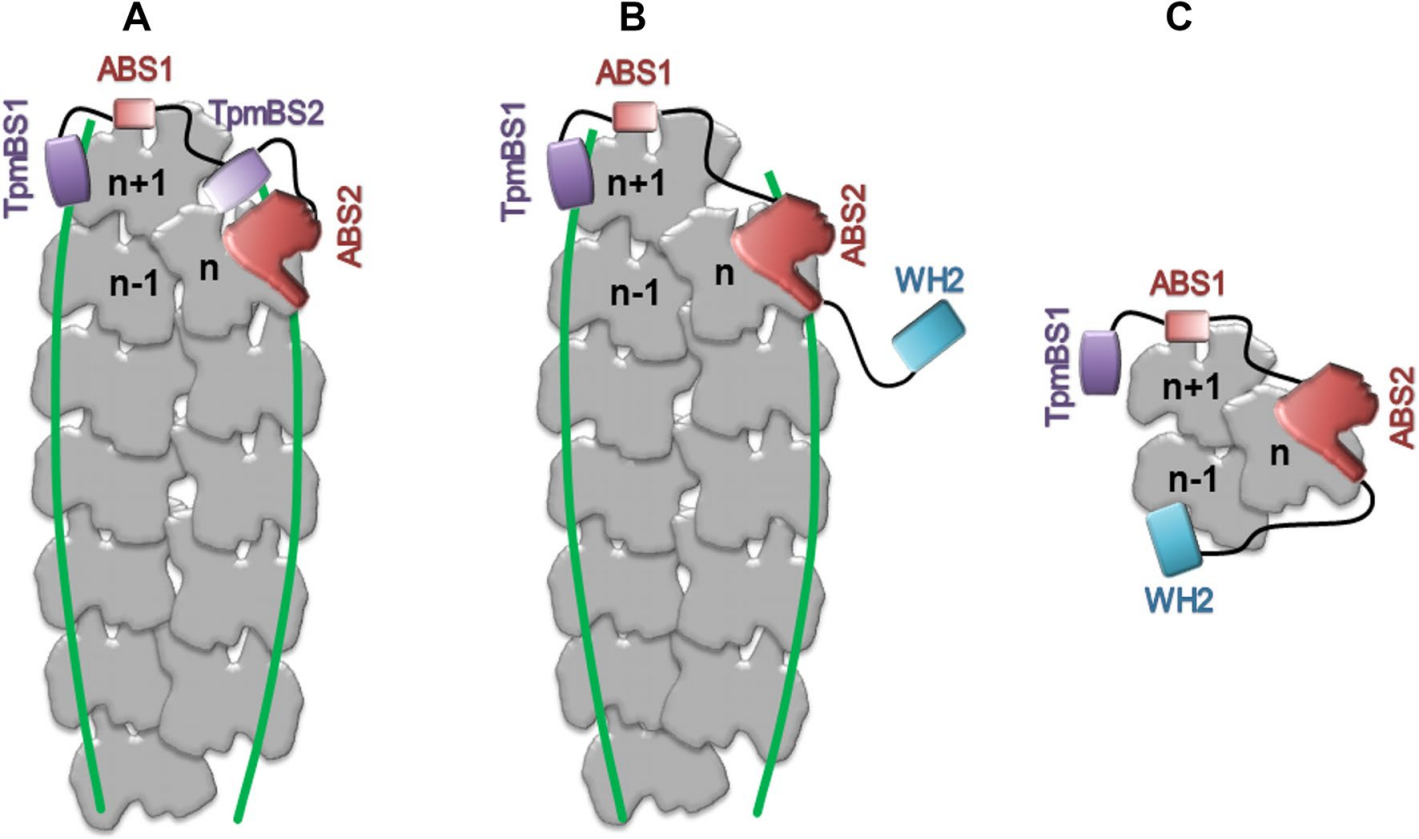
Schematic illustration of pointed end capping by Tmod (**a**) or Lmod (**b**) and formation of actin polymerization nuclei by Lmod (**c**). Actin subunits and tropomyosin chains are marked with grey and green, respectively. TpmBS1 and TpmBS2—tropomyosin-binding sites 1 and 2; ABS1 and ABS2—actin-binding sites 1 and 2, WH2—WASP homology 2 domain. The drawing is based on the models proposed in (Chen et al. 2015; Rao et al. 2014). (Color figure online)

Tmod and Lmod are homologous proteins containing tropomyosin and actin-binding domains (Fig. 3). The N-terminal half of Tmod comprises two tropomyosin binding sites (TpmBS1 and TpmBS2) and a tropomyosin-dependent actin binding site (ABS1). The second actin-binding site (ABS2) is localized in the C-terminal, globular, leucine-reach domain of Tmod [reviewed in (Colpan et al. 2013; Fowler and Dominguez 2017)]. The alternating tropomyosin and actin-binding sites allow Tmod to cap the thin filament pointed end (Fig. 3a) and very efficiently inhibit the elongation of actin filament. Tropomyosin-binding sites interact with N-terminal segments of individual tropomyosin molecules associated with actin filaments, which enables the helix contained in ABS1 to bind across subdomain 2 and 4 of the actin subunit exposed on the pointed end of one strand of the filament (subunit n + 1). ABS2 wraps around the filament and binds to subdomains 1 and 2 of the actin subunit located on top of the second actin strand (subunit n) (Kostyukova et al. 2007; Rao et al. 2014).

In contrast, the N-terminal domain of Lmod contains only one conserved tropomyosin-binding site followed by less conserved ABS1. The C-terminal half of Lmod contains a highly conserved leucine-reach repeat, which harbors ABS2, followed by a proline-reach domain and WH2 domain (Wiscott-Aldrich syndrome protein homology 2). The crystal structure of Lmod2’s C-terminal half in complex with actin monomer in turn combined with prediction of the ABS1 position on the adjacent subunit led to a proposed model of the Lmod binding modes to actin filment (Chen et al. 2015). This model supports the view that the two actin binding sites in Lmod bind actin subunits in a manner similar to Tmod (Fig. 3b). The presence of the WH2 domain, which binds to the cleft between actin subdomains 1 and 3, makes Lmod capable of binding to the third actin subunit (Fig. 3c). Thus, Lmod has the capacity to nucleate actin polymerization, which masks its pointed end capping ability (Chen et al. 2015; Chereau et al. 2008; Tsukada et al. 2011).

Two possible mechanisms of Lmod and Tmod activities in myocytes were proposed. According to one model, Lmod first nucleates new filaments and then it dissociates leaving the actin nucleus for polymerization. The pointed ends of the newly formed filaments compete for Tmod bound to old filaments, causing Tmod’s dissociation and elongation of the pre-existing filaments (Chereau et al. 2008; Fowler and Dominguez 2017). The second model assumes that in mature myocytes, Lmod competes with Tmod and transiently displaces it from the pointed end. Because the second tropomyosin-binding site in Lmod is missing, it cannot inhibit polymerization of actin at the pointed end as much as Tmod; therefore Lmod acts as a “leaky cap” and the thin filaments elongate. Once Tmod is re-bound, the elongation is inhibited (Ly et al. 2016; Tsukada et al. 2010). The length of the thin filaments can therefore be regulated by a competition for the pointed end between Lmod and Tmod. Both models agree with the crystal structure of Lmod2 C-terminal region in complex with actin, which suggests that Lmod can participate in actin filament nucleation as well as the pointed end elongation (Chen et al. 2015). Whichever mechanism takes place in the cell, the growth at the pointed end must depend on expression levels of Lmod and Tmod, which vary in different tissues and developmental stages [for a review see (Colpan et al. 2013; Fowler and Dominguez 2017)]. Because tropomyosin isoforms affect the affinity of Tmod and Lmod isoforms individually (Colpan et al. 2013; Kostyukova 2007; Tsukada et al. 2010; Yamashiro et al. 2014), they have the potential to orchestrate the filament dynamics at the pointed end.

The exchange of actin subunits at the pointed ends is accelerated by ADF/cofilin, a family of proteins that bind to the sides of actin filaments, sever the filaments and accelerate depolymerization. In striated muscle, the activity of muscle-specific cofilin-2 is necessary for the maintenance of the sarcomere structure and regulation of the precise length of the thin filaments (Agrawal et al. 2012; Kremneva et al. 2014). Most tropomyosin isoforms compete with cofilin for binding to the actin filament, and thus attenuate severing and depolymerization rates [reviewed in: (Kuhn and Bamburg 2008)]. It is interesting to note that in the absence of tropomyosin, cofilin binds cooperatively along the whole filament, but it accumulates near the pointed end on tropomyosin-covered filaments (Jansen and Goode 2019; Kremneva et al. 2014). Binding of cofilin, Tmod and Lmod in one region of the sarcomere may create conditions for fine tuning the thin filament length; however, mechanisms of cooperative interactions between these proteins are not well understood.

## Effects of disease-causing mutations on interactions of tropomyosin with actin

As described earlier, tropomyosin is the crucial determinant of the Ca^2+^-dependent regulation of actin-myosin interactions, because the position it assumes on the thin filament either blocks or exposes myosin-binding sites. High resolution models of F-actin-Tpm (von der Ecken et al. 2015) and F-actin-Tpm-myosin S1 (Behrmann et al. 2012) provide details of the interactions within the thin filament. These can be experimentally verified by the analysis of tropomyosin mutants and their effects on actin binding parameters, which shed light on the importance of specific regions of tropomyosin in stabilization or destabilization of the activation states.

Several mutations were identified in actin-binding sites which electrostatically interact with actin. Charge reversal or neutralization within these sites was predicted to affect tropomyosin affinity for actin. Using in silico analysis of the binding energy, Orzechowski and colleagues predicted relative affinity of tropomyosin for actin in the B-state (Orzechowski et al. 2014). Tpm1.1 carrying hypercontractile substitutions Asp58His or Glu62Gln (Fig. 1b) was predicted to decrease actin affinity and destabilize the B-state, which shifts the filament into a more active state. If stabilization of the B-state were the determinant of the hypocontractile phenotype, one would expect that mutations associated with muscle weakness will bind to actin in the B-state with higher affinity. Neither energy landscape analysis, nor equilibrium binding experiments confirmed this prediction. Binding energy calculations and results of co-sedimentation of F-actin with Tpm1.1 carrying hypocontractile substitutions Arg167His or Arg244Gly showed decreased actin affinity and B-state destabilization (Orzechowski et al. 2014; Robaszkiewicz et al. 2012). In myopathy patients, the mutations were found in the *TPM3* gene encoding Tpm3.12; therefore the substitutions Arg167His and Arg244Gly introduced in Tpm1.1 were counterparts of the substitutions in Tpm3.12 (Fig. 1b). Because of the high similarity between these isoforms, in the computational and in vitro studies Tpm1.1 was used as a model protein. Such an approach might be questionable because of the 25 amino acid differences between Tpm1.1 and Tpm3.12. To check whether the disease-causing mutations have a greater impact on actin affinity than the isoform-dependent substitutions, we have recently analyzed the effects of Arg167His in recombinant Tpm1.1 and Arg168His in Tpm3.12 on actin affinity. The comparison of wild type and mutant tropomyosins confirmed the profound effects of the myopathy-causing mutations on tropomyosin affinity for the actin filament (Moraczewska, unpublished results). In addition, the substitution Arg167Cys in Tpm1.1 (Robaszkiewicz et al. 2012) and Arg168Cys in Tpm3.12 (Yuen et al. 2015) were shown to reduce actin affinity of both isoforms, which supports the view that Arg167(168) is an evolutionary conserved consensus site crucial for stabilization of tropomyosin interactions with the actin filament (Barua et al. 2011). It is surprising though, that despite weakening of the ionic interactions between mutant tropomyosin and the charged residues exposed on the actin surface (Arg147 and Lys328), the substitution of the consensus site Glu62 with Gln does not change Tpm1.1 affinity for actin (Farman et al. 2018; Gupte et al. 2015), which demonstrates that the Glu62Gln has a local effect and does not contribute to the overall tropomyosin-actin affinity.

Mutations located at the coiled coil interface can disturb local or global structure of tropomyosin and affect interactions with actin. Two mutations in Tpm1.1—Ile92Thr and Ala95Val, located close to each other in *a* and *d* positions of the heptapeptide repeat, have been linked to DCM and HCM, respectively (Karibe et al. 2001; Redwood and Robinson 2013), which implies that the mutations have opposite functional effects. One of the differences is that Ile92Thr reduces binding of Tpm1.1 to actin in the absence of Tn or in the presence of Tn + Ca^2+^ (C-state), whereas Ala95Val slightly increases the affinity (Sliwinska et al. 2018). Structural effects of Ile92Thr are unknown, but Ala95Val was shown to decrease the content of the α-helix (Wang et al. 2011) and to possibly reduce overall flexibility of Tpm1.1 (Zheng et al. 2016). Because in the Tpm1.1 sequence, Val95 precedes a cluster of three Glu residues (96–98) which harbors Glu97, directly interacting with actin (Li et al. 2011), the conformational changes induced by the substitution may bring this segment of Tpm1.1 closer to the positively charged patch on actin thereby increasing the affinity (Sliwinska et al. 2018).

A number of cardiomyopathy-linked mutations are located in *g* and *e* positions of the heptapeptide repeat. Extensive studies on the Asp175Asn (*g* position) and Glu180Gly (*e* position) mutations associated with HCM demonstrated profound structural changes in Tpm1.1, such as decreased α-helical content (Golitsina et al. 1997; Ly and Lehrer 2012; Wang et al. 2011), increased overall bending flexibility and local flexibility around Glu180 (Li et al. 2012), and decreased thermal stability of the Tpm1.1-actin complex (Kremneva et al. 2004). These conformational changes reduce tropomyosin affinity for actin in the C-state (Golitsina et al. 1997), which is probably due to the localization of the mutation sites near the residues Glu181and Glu184 directly interacting with basic residues on actin (Li et al. 2012). Results of the in vitro analyses were confirmed in transgenic mice models, which demonstrated an increased activation of the thin filament through enhanced Ca^2+^ sensitivity of steady-state force (Muthuchamy et al. 1999; Prabhakar et al. 2001).

Two mutations—Glu40Lys and Glu54Lys, which reverse charge in *e* positions of Tpm1.1, result in DCM (Olson et al. 2001). Reconstitution of the thin filaments in cardiac fibers with both Tpm1.1 mutants led to a decreased force generation at systole (Bai et al. 2012) and reduced sensitivity to [Ca^2+^] (Mirza et al. 2005). In vitro studies revealed that the mechanism underlying the development of the DCM phenotype linked to these mutations is not simple. While the substitution Glu54Lys had no effect on the equilibrium of the On–Off transition, Glu40Lys decreased the equilibrium of the On–Off transition (Mirza et al. 2007). This explains the observation that at less than saturating [Ca^2+^] Glu40Lys, but not Glu54Lys, reduced maximal activation of actin-activated myosin S1 ATPase (Mirza et al. 2005).

Both mutations affected the stability of the coiled coil; however, independent studies were not mutually consistent. In one study, none of the mutations changed the α-helical content, but both mutations caused decreased thermal stability of N-terminal domain of Tpm1.1 in the absence and presence of F-actin (Mirza et al. 2007). In another report, Glu54Lys was shown to increase thermal stability of Tpm1.1 (Rajan et al. 2007). In spite of the discrepancy, the studies showed that the substitutions were not neutral to tropomyosin structure.

Glu40Lys and Glu54Lys are located in tropomyosin’s β-band and α-band, respectively. Conformational changes caused by the Glu54Lys substitution reduced Tpm1.1 affinity for actin alone, but the substitution Glu40Lys had no affect. In turn, the affinity of Tpm1.1 for F-actin-myosin S1 (M-state) was decreased by Glu40Lys, but not by Glu54Lys. This observation was thought to reflect a rolling of the tropomyosin coiled coil to switch between the activation states (Mirza et al. 2007). In contrast, the substitution Ala155Thr located in the β-band was shown to reduce actin affinity by about two fold in both the B- and the C-state (Robaszkiewicz et al. 2012). Such effects could also be explained by the alternative shifting mechanism of tropomyosin between the different regulatory positions. These apparently contradictory conclusions can be reconciled by a mutation-dependent change in local tropomyosin twisting. Molecular dynamics studies on the conformation of Tpm1.1 suggested the small impact of Glu40Lys mutation to tropomyosin-actin association, but significant effects on side chain interactions between tropomyosin chains, which increased tropomyosin flexibility (Farman et al. 2018). If sliding of the tropomyosin cable is accompanied by local twisting during regulatory transitions, Glu40 located in the β-band could be shifted towards the actin surface, hence the effect of Glu40Lys on tropomyosin affinity in the M-state. On the other hand, Ala155Thr is located in the *a* position of the heptapeptide repeat within the core of the coiled coil and was shown to decrease thermal stability of Tpm1.1 (Robaszkiewicz et al. 2015). It is possible that destabilization of the coiled coil leads to increased flexibility, which equally affects tropomyosin-actin interface in both activation states.

In summary, the effects of pathogenic mutations which have been analyzed in the context of structural data demonstrate a high complexity of the tropomyosin-dependent mechanisms of the filament activation.

## Mechanisms of Ca-dependent regulation of actin–myosin interactions—lesson learned from the studies on mutant tropomyosin variants

As suggested by hyper- and hypocontractile effects of mutations in *TPM1* and *TPM3*, distinct regions of tropomyosin, which are distributed along the whole length of the molecule, contribute to the Ca-dependent regulation of actin-myosin interactions. Enhanced actin-myosin ATPase rate at activating [Ca^2+^], diminished relaxation at low [Ca^2+^], increased Ca-sensitivity observed as a shift of Ca-dependence towards lower [Ca^2+^], and higher speed of the thin filaments in in vitro motility assays are the functional effects that are most frequently reported for HCM mutants in Tpm1.1 [e.g. (Chang et al. 2005; Gupte et al. 2015; Ly et al. 2019; Wang et al. 2011)]. The hypercontractile effects have been attributed to destabilization of the B-state (Farman et al. 2018; Mathur et al. 2011; Orzechowski et al. 2014; Zheng et al. 2016).

In contrast, decreased acto-myosin interactions underlie hypocontractile phenotypes associated with substitutions in Tpm1.1 and Tpm3.12, causing DCM and congenital myopathies. Various in vitro assays as well as fiber experiments show that this phenotype manifests itself through reduced activation of actin-myosin ATPase with preserved inhibitory function, decreased Ca-sensitivity, and reduced motility of the thin filaments (Farman et al. 2018; Gupte et al. 2015; Ly et al. 2019; Marston et al. 2013; Moraczewska et al. 2019; Robaszkiewicz et al. 2012; Yuen et al. 2015). However, hypocontraction cannot be directly linked to stabilization of the inhibitory state, because the mutations do not show a regular pattern in terms of changes in actin affinity. As discussed above, some mutants increase their affinity for actin, but most bind to actin in the absence or presence of Tn (± Ca^2+^) with decreased affinity (Gupte et al. 2015; Orzechowski et al. 2014; Robaszkiewicz et al. 2012; Yuen et al. 2015).

Because Tn is a sensor of Ca^2+^ levels in sarcoplasm, one can expect that mutation-dependent changes in the thin filament response to Ca^2+^ signals are due to structural alterations in the Tn complex brought about by defects in interactions between Tn and mutant tropomyosins. Changes in orientation of tropomyosin upon binding of Tn were demonstrated by tracking excimer fluorescence of pyrene attached to Cys190 (Ishii and Lehrer 1990). This method was used to show that myopathy-linked single amino acid substitutions (Leu99Met, Ala155Thr, Arg167Cys/Gly/His, Lys168Glu and Arg244Gly) altered conformation in the central part of tropomyosin, which was sensitive to Tn binding. The effect was larger when the site of the substitution was closer to the binding region of the Tn core domain. This effect correlated with the reduction of Tn affinity for mutated tropomyosin (Robaszkiewicz et al. 2012).

Conformational changes in Tn core domain can be followed by FRET between donor and acceptor pairs located in interacting regions of cardiac TnI and TnC. After Ca^2+^ saturation the interprobe distances decrease, indicating binding of the TnI regulatory region to a hydrophobic pocket within the N-terminal domain of TnC (Xing et al. 2009). The TnI–TnC interaction is disturbed by DCM-linked substitution Lys15Asn in Tpm1.1, which increases the interprobe distances. In contrast, the Arg21His mutation, which associated with HCM, has no effect on the measured distances. The conformational changes in Tn core domain correlate with acto-myosin ATPase rate, which for the Lys15Asn mutant is lower compared to the wild type Tpm1.1 (Ly et al. 2019). Thus, structural changes associated with DCM-causing substitutions in tropomyosin are propagated to the N-terminal domain of TnC. Lys15 is far from the central region of tropomyosin, involved in binding of the Tn core domain, and is at the *a* position within the overlap between neighboring tropomyosins and TnT. Conformational changes induced by the substitution of Lys15Asn decrease stability of the coiled coil (Colpan et al. 2016a) and can be propagated through TnT to the core domain containing TnC. Involvement of TnT in the transmission of structural changes along the thin filament is supported by the observation that the DCM-causing mutation Asp230Asn decreases FRET distances measured between residues embedded in the C-terminal segment of Tpm1.1 and cardiac TnT (McConnell et al. 2017). The structural correspondence between tropomyosin regions affected by cardiomyopathy mutations and cardiac TnC has been demonstrated by fluorescence of ANS (anilino-naphtahlene-6-sulfonic acid) attached specifically to residue 53 (Thr53 replaced with Cys). At saturating Ca^2+^ concentrations the intensity of ANS fluorescence in the thin filaments reconstituted with Tpm1.1 HCM mutants (Glu62Gln, Leu185Arg, Ser215Leu, Met281Thr) increased much above the intensity observed in the presence of wild type Tpm1.1. This was in contrast to DCM-causing mutations Asp84Asn and Asp230Asn, which did not alter the environment of the probe (Greenfield and Fowler 2002; Gupte et al. 2015).

The above mentioned experimental data support the idea that defects in structural connection between tropomyosin and different subunits of Tn complex produced by the mutations in tropomyosin cause changes in response of the thin filament to Ca^2+^ signalling. Other experimental evidence shows that cooperativity of the myosin-induced transition from the C- to M-state is another factor that contributes to the thin filament activation. Strongly bound myosin heads cooperatively increase tropomyosin binding to actin in the M-state (Eaton 1976). In the absence of tropomyosin, the actin-myosin ATPase activity increases linearly with increased concentration of myosin head (S1) concentration, but in the presence of tropomyosin this dependence departs from linearity—at low S1 concentration the ATPase is below the activity of unregulated actin and at high S1 concentrations the ATPase is potentiated, which means it is far above the ATPase observed for actin alone. The presence of Tn+Ca^2+^ facilitates the potentiation process (Lehrer and Morris 1982). Myopathy-causing mutations located in the actin-binding period 5 (Arg168Cys/His/Gly and Lys169Glu), have been shown to decrease cooperativity of C- to M-state transition by increasing the concentrations of S1 required for potentiation both in the presence and absence of Tn+Ca^2+^. Other mutations associated with myopathies, but located in different tropomyosin regions (Leu100Met, Ala156Thr and Arg245Gly), do not affect the cooperativity of the C- to M-state transition (Robaszkiewicz et al. 2012). The results agree well with earlier studies showing that deletion of the actin-binding period 5 has the most severe effect on cooperativity of the interactions between the thin filaments and myosin (Hitchcock-DeGregori et al. 2002). It is noteworthy that the mutations were tested in Tpm1.1; therefore the results shed light on the structure–function relationships of this isoform. In patients the mutations were found in Tpm3.12, so that it remains to be established whether in Tpm3.12 these substitutions have comparable effects on the S1-induced potentiation and are one of the determinants of the hypocontractile phenotypes.

In conclusion, studies on tropomyosin mutations linked to skeletal and cardiac muscle diseases deliver valuable data, which support the idea that tropomyosin uses multiple mechanisms to control thin filament activation.

## Mutations in Tpm1.1 and Tpm3.12 as tools in the studies on the tropomyosin-dependent regulation of the thin filament dynamics at the pointed end

As mentioned above, the N-terminal region of tropomyosin interacts with Tmod and Lmod at the pointed end of the thin filament (Fig. 1c). Using N-terminal peptides of different lengths, Kostyukova and her colleagues established that the segment of Tpm1.1, which interacts with Lmod2, comprises the first 21 amino acids and is longer than the segment interacting with Tmod1 (amino acids 1–14) (Colpan et al. 2016a; Greenfield and Fowler 2002). Within the N-terminal segment, Tpm1.1 differs from Tpm3.12 only in two amino acids (Fig. 1a). It is not known yet whether these changes cause significant differences in Tmod and Lmod binding to the thin filament. So far, the effects on binding of Tmod or Lmod of four disease-associated mutations in the actin-binding period 1 and two mutations in the inner regions of tropomyosin (actin-binding periods 3 and 5) have been studied. These data revealed mechanisms which allow tropomyosin to efficiently regulate elongation at the pointed end of the thin filament.

The Met9Arg substitution in Tpm3.12 and analogous Met8Arg substitution in Tpm1.1 have been linked to hypocontractile phenotypes—NM and DCM, respectively (Laing et al. 1995; Lakdawala et al. 2012). The presence of Met8Arg substitution in a peptide comprising fourteen N-terminal residues of Tpm1.1 abolishes interactions with the N-terminal fragment of Tmod (Greenfield and Fowler 2002). Met8 is located in the core of the coiled coil (*a* position of the heptapeptide repeat); therefore substitution with Arg greatly destabilizes the structure of the N-terminus (Moraczewska et al. 2000), which distorts the interactions with Tmod. In contrast, the substitution Ala4Val in Tpm3.12, which is associated with CFTD (Marttila et al. 2014b), does not interfere with binding of full length Tmod1 and only mildly reduces Tmod1’s ability to inhibit the thin filament elongation at the pointed end (Moraczewska et al. 2019). Ala4 is at the *c* position of the heptapeptide repeat, and therefore it is located outside the core of the coiled coil. Modeling of the full length Tpm3.12 by molecular dynamics did not detect significant differences in flexibility of the N-terminal segment between wild type and the Ala4Val mutant. Hence interactions with Tmod1 seem not to be disturbed (Moraczewska et al. 2019).

Two cardiomyopathy mutations, Lys15Asn and Arg21His, also destabilize the local secondary structure of the N-terminal segment of Tpm1.1. Although Lys15 is located in the core of the coiled coil (*a* position) and Arg21 is outside the core (*g* position), mutation of Arg21 leads to a more dramatic loss of the helical content (Colpan et al. 2016b; Ly et al. 2018). These structural changes are due to the loss of interchain salt bridges formed by Lys15 and Arg21 (Ly et al. 2019). The DCM-causing Lys15Asn decreases the affinity of Tpm1.1 to the tropomyosin-binding site of Lmod2 and both tropomyosin-binding sites of Tmod1. Defects in the interactions between the N-terminal segment of the mutant tropomyosin and the isolated tropomyosin-binding sites of Lmod2 and Tmod1 result in significantly reduced binding of full length proteins and impaired abilities to inhibit elongation of the pointed end (Colpan et al. 2017). The HCM-linked mutation Arg21His causes a severe decrease in the affinity for the isolated tropomyosin binding site of Lmod2 and diminishes the ability of Lmod2 to inhibit elongation of the pointed end (Ly et al. 2019). Unlike Lys15Asn, the substitution Arg21His does not alter the inhibition of the pointed end elongation by Tmod1 (Ly et al. 2019). The differential effects on Tmod and Lmod binding and activity affect the dynamics of the pointed end and can contribute to the development of the hypo- or hypercontractile phenotypes in cardiomyocytes.

Interestingly, the substitution of Arg91Cys in Tpm 3.12 reduces the actin affinity and pointed end elongation and leads to muscle weakness and type 1 fiber hypotrophy (Marttila et al. 2014b). Because Arg91 is located in actin-binding period 3, it is not involved in direct Tmp3.12-Tmod1 interactions. However, Arg91 is a conserved residue which interacts electrostatically with actin (Li et al. 2011). Molecular dynamics simulations predicted that charge reduction in this site can increase the distance between Tpm3.12 and the axis of the filament (Moraczewska et al. 2019). Such a shift may distort the interface between Tpm3.12 and Tmod1, leading to reduced binding. In the muscle, destabilization of interactions with Tmod may cause increased Tmod dissociation from the pointed end and depolymerization of the filaments. Shorter thin filaments were observed in fibers of a nemaline myopathy patient carrying Arg168His mutation in *TPM3*. The length was reduced only within the nebulin-free extension at the pointed end, which suggests decreased affinity for Tmod4 (Ochala et al. 2012). In terms of binding to actin, Arg168 is an equivalent of Arg91, though it is located in actin-binding period 5 (Li et al. 2011). If breaking contacts with actin varies the position of Tpm3.12, the affinity for Tmod will drop as observed for the Arg91Cys mutant.

In conclusion, the available data suggests that tropomyosin controls functions of Tmod and Lmod, the pointed end-binding proteins, by specific interactions and by changing the interface between the N-terminal region of tropomyosin and the proteins binding at the pointed end. Further analyses of Tpm1.1 and Tpm3.12 in combination with Tmod and Lmod isoforms are needed to uncover the molecular mechanisms regulating the dynamics at the pointed ends of the thin filaments in different types of muscle.

## Abbreviations

Tpm: Tropomyosin
Tn: Troponin complex
Tmod: Tropomodulin
Lmod: Leiomodin
HCM: Hypertrophic cardiomyopathy
DCM: Dilated cardiomyopathy
NM: Nemaline myopathy
CFTD: Congenital fiber type disproportion
EM: Electron microscopy
FRET: Förster resonance energy transfer
ANS: Anilino-naphtahlene-6-sulfonic acid
